# Professor E. Lloyd Howard

**Published:** 1881-11

**Authors:** 


### OBITUARY.

*Professor Edward Lloyd Howard, M. D.*—

It is our sad and painful duty to record the death of Prof. E. Lloyd Howard, M. D. who died suddenly September 5, 1881, of uræmic apoplexy.

At the time of his death Prof. Howard occupied the chair of Chemistry and Materia Medica in the Baltimore College of Dental Surgery, the duties of which he performed with rare ability and in a satisfactory manner to the many classes which have annually been in attendance, during his long connection with the institution, extending over a period of thirteen years. Prof. Howard at the time of his death was also President of the Maryland State Board of Health, and Resident Physician at the Marine Hospital, Port of Baltimore.

He was born in Baltimore City, January 14th 1837, and was the son of the late Charles Howard, and grandson of Gen. John Eager Howard, of revolutionary fame. Dr. Howard's mother is a daughter of Francis S. Key, the author of the "Star Spangled Banner."

After a careful training and a Collegiate Course, Dr. Howard studied medicine and graduated at the University of Maryland School of Medicine, and at once began practice in Baltimore, but on the breaking out of the civil war went South.

The distinction which Dr. Howard had won among his classmates at the University was recognized in Richmond when he went before the board of examiners for the place of assistant surgeon in the Confederate army. The board passed him, and in their report stated the high opinion in which they held him for his medical acquirements, and that he was even capable of filling the position of full surgeon. In consequence of this the Secretary of War sent him a commission making him surgeon, instead of assistant surgeon. Dr. Howard discharged his duties in the Confederate service on numerous fields and in different hospitals with marked success, and with a zeal and interest that never flagged. At the close of the war he returned to Baltimore and once more entered upon the practice of his profession.

During the small pox epidemic, an epoch in the history of Baltimore, he volunteered his services, and went fearlessly among the sufferers of the dread disease. It was during this trying time that the late Mr. Wm. Schley was taken to the Marine Hospital. Dr. Howard was untiring in his attentions upon him. After the resignation of Dr. J. S. Conrad the place of resident physician at the Marine Hospital was given to Dr. Howard, his quiet demonstration of his fitness for the place having been most striking. This appointment was made about five years ago, and about two years ago Dr. Howard was further honored by being made President of the Maryland State Board of Health, a position which he has filled to the satisfaction of all. Dr. Howard was all through his career a hard student, kept ahead of the times in many things and fully abreast of them in all others. During the prevalence of typho-malarial fever on Fell's Point in 1876 he was very active, and did much to alleviate the sufferings of the victims of the disease. Dr. Howard had no fear of the contagious diseases, such as yellow fever, small-pox, typhus fever, &c., and went among them without reserve, though he adopted the usual precautions to prevent taking them. Many are to-day living to testify to his fearless discharge of duty and his skillful treatment. He was one of the incorporators of the College of Physicians and Surgeons, and has been prominent in its work since it started, about five years ago. Last year he occupied the chair of medical jurisprudence and toxicology in the college, and at the last meeting of the faculty was elected to the chair of materia medica. He had also occupied the chair of chemistry.

In 1868 Prof. Howard entered the Faculty of the Baltimore College of Dental Surgery as Demonstrator of Anatomy. In 1869 he was selected Professor of Anatomy, and in 1874, Professor of Chemistry and Materia Medica, which he held at the time of his death.

Probably the greatest compliment paid him was his appointment on the yellow fever committee sent out by the United States government in 1878 to investigate the causes of and make reports on the devastation by that fearful disease in the Southern cities and the Mississippi Valley. Dr. Howard, in connection with the other gentlemen of that commission, made his report at the meeting of the American Health Association in Richmond, Va., in November, 1878. The meeting drew together the learned men of the profession from all parts of the country, and the convention was of the most satisfactory character. For so young a man to hold the place he did in that convention was an indorsement of his acquirements and ability.

Prof. Howard was a man of rare attainments, and one of the most widely known and popular members of the medical profession in Baltimore City.

His death is a loss to the community, as it is to a large circle of friends and connections, and sincere expressions of sorrow were heard from all with whom he was acquainted when the news of his sudden death quickly spread throughout the city.

The funeral took place on Sept. 7th, from the residence of his mother, No. 53 Cathedral Street, and was numerously attended by the medical and dental professions, city officials and many friends. The pall-bearers were Mayor Latrobe, Dr. James A. Steuart, Dr. L. McLane Tiffany, Dr. Thomas Opie, Dr. F. J. S. Gorgas, Dr. F. C. Chatard, Dr. O. F. Coskery and D. C. Clark.

The body was placed in a handsome cloth-covered casket, furnished by Messrs. H. W. Jenkins & Sons, bearing a plate with the name and age of the deceased, and was literally covered with the most exquisite crosses, anchors, pillows, etc.

The funeral services were conducted by Rev. Augustus P. Stryker, of St. Barnabas P. E. Church. At the conclusion of the services the cortege removed to Garrison Forest grave-yard, near Owings' Mills, where the interment was made, Revs. W. F. Lockwood and W. S. Jones officiating.

At a meeting of the Faculty of the Baltimore College of Dental Surgery, held in the College Building September 22d, 1881, the following resolutions were unanimously adopted:

### IN MEMORIAM.

*Whereas* the unexpected close of a prominent and useful life in the death of PROFESSOR EDWARD LLOYD HOWARD, M. D., who for the past thirteen years has been an honored member of this Faculty, renders it obligatory upon his colleagues to express their high appreciation of his exalted character and professional worth, and to place on record their regret for his loss, and their sense of his merit:

*Resolved*, That in the death of Prof. Howard, our College has been deprived of an efficient teacher and this Faculty of one of its most cherished members, who, by his labors and sacrifices in behalf of medical scisence, his courage and devotion amidst the greatest dangers of contagion and death, his lofty aspirations for the advancement of the science to which he devoted his life, and his untiring efforts in behalf of afflicted humanity has merited the highest meed of praise and afforded an example worthy of imitation by his professional brethren, and which entitled him to the respect and gratitude of this community.

*Resolved*, That the Dean be directed to present to the family of the deceased a copy of these resolutions, together with a life-size portrait, with assurances of our sincere sympathy in their bereavement; also

*Resolved*, As a further mark of respect, that a similar portrait be hung in the Faculty Room of the College.

F. J. S. Gorgas,
Thos. S. Latimer,
R. B. Winder,
*Committee.*

At a meeting of the students in attendance on the summer course of the Baltimore College of Dental Surgery, the following resolutions were adopted:

*Whereas*, it has pleased Almighty God, the giver and taker of life, to bereave us in the death of our esteemed and beloved Professor, E. Lloyd Howard, we, the students of the Baltimore College of Dental Surgery, in body assembled, do feel deeply grieved at our loss, and sympathize with his family in their affliction.

*Resolved*, That an expression of our feelings and regard be tendered to the family, and a copy published in the Baltimore *Sun.*

L. P. Dotterer,

C. A. Slocum,

J. S. Bizzell,

*Committee.*

M. Gist Sykes,

*Secretary.*

